# Working Conditions, Socioeconomic Factors and Low Birth Weight: Path Analysis

**DOI:** 10.5812/ircmj.11449

**Published:** 2013-09-05

**Authors:** Zohreh Mahmoodi, Masoud Karimlou, Homeira Sajjadi, Masoumeh Dejman, Meroe Vameghi, Mahrokh Dolatian

**Affiliations:** 1 Social Determinant of Health Research Center, University of Social Welfare and Rehabilitation Sciences, Tehran, IR Iran; 2 Department of Midwifery, Shahid Beheshti University of Medical Sciences, Tehran, IR Iran

**Keywords:** Socioeconomic Factors, Low Birth Weight, Health

## Abstract

**Background:**

In recent years, with socioeconomic changes in the society, the presence of women in the workplace is inevitable. The differences in working condition, especially for pregnant women, has adverse consequences like low birth weight.

**Objectives:**

This study was conducted with the aim to model the relationship between working conditions, socioeconomic factors, and birth weight.

**Patients and Methods:**

This study was conducted in case-control design. The control group consisted of 500 women with normal weight babies, and the case group, 250 women with low weight babies from selected hospitals in Tehran. Data were collected using a researcher-made questionnaire to determine mothers’ lifestyle during pregnancy with low birth weight with health-affecting social determinants approach. This questionnaire investigated women’s occupational lifestyle in terms of working conditions, activities, and job satisfaction. Data were analyzed with SPSS-16 and Lisrel-8.8 software using statistical path analysis.

**Results:**

The final path model fitted well (CFI =1, RMSEA=0.00) and showed that among direct paths, working condition (β=-0.032), among indirect paths, household income (β=-0.42), and in the overall effect, unemployed spouse (β=-0.1828) had the most effects on the low birth weight. Negative coefficients indicate decreasing effect on birth weight.

**Conclusions:**

Based on the path analysis model, working condition and socioeconomic status directly and indirectly influence birth weight. Thus, as well as attention to treatment and health care (biological aspect), special attention must also be paid to mothers’ socioeconomic factors.

## 1. Background

In recent years, with socioeconomic changes in the society, women’s role has also significantly changed, and their share of the labor market has increased dramatically, comprising 42% of the working population, they are now an essential part of the national economy (1). In Iran too, as in most developing countries, in tandem with socioeconomic changes, women’s desire to work and career opportunities have increased (2). However, due to gender differences, women are faced with physiological changes in life different from men that can be affected by their conditions, environment, and type of work. Among these are menstruation, pregnancy, and child care (1).

Most women’s desire to work out of home often means delaying marriage and pregnancy until older years, which affects the person’s health and ultimately the society, as well (3). In addition, working conditions including stress, long standing position, contact with chemical products could have undesirable consequences such as spontaneous abortion, pre-term delivery, low birth weight, and infant deformities. Also, evidence has been published revealing adverse effects of occupational stress on fetal growth and development (4). According to conceptual framework, working conditions and health inequalities are related to each other through a number of psycho-social, behavioral and psychological mechanisms. Risk factors are present in four main categories; physical, chemical, ergonomic, and psycho-social such as; physical hazards, chemical hazards, repetitive movements, hard and intensified physical work, shifts, or lack of supervision (5). Therefore, biological, psycho-social, and social differences, together with exposure to occupational hazards, create a specific gender pattern for occupational health problems (1). However, in recent decades, despite the changes mentioned, studies related to occupation and occupational factors associated with pregnancy outcomes have reduced in number (6).

The effect of working conditions on adverse pregnancy outcomes is a controversial subject. Most studies on the impact of working conditions on pregnancy outcomes have not identified equal working conditions as a risk factor, and also, a number of studies have not found any relationship between working conditions and adverse pregnancy outcomes, including low birth weight. On the other hand, several reviews and meta-analyses have reported the impact of working duration, work shift, and job stresses on these outcomes (7). Adverse pregnancy outcomes such as low birth weight and preterm birth are extremely important and dangerous factors affecting neonatal mortality and morbidity (6). Birth weight is an important determinant of a child’s growth and development, and indicator of his future physical health (8). From the stand point of public health, mean birth weight in a society is a sign of quality of the extensive healthcare services, availability of nutrition and care to mothers, as well as being a useful benchmark for monitoring the quality of prenatal care and intrauterine growth. Growth disorders in this period become an important factor in low birth weight, increased prenatal mortality and morbidity in infancy and in adulthood (9, 10). Birth size is indicative of two factors of gestational age and fetal growth. Therefore, it should be considered with regards to the gestational age. Otherwise, the size increase that occurs with aging may interfere in the expression of fetal growth and maturity (11). According to the World Health Organization (WHO), birth weight less than 2500 grams is considered Low Birth Weight (LBW)1 (12). Compared to normal infants, LBW infants are more exposed to such risks as cerebral palsy, mental retardation, incidence of neurological impairments, respiratory diseases, sudden death syndrome, and complications due to hospitalization in intensive care units (12-16). In addition to the physical and psychological problems, maintenance and treatment costs of these infants are six times as other normal infants (17).

But, despite achievements in health in recent years, the continued high incidence of low birth weight is indicative of the importance of the need to attend to its influencing factors. The socioeconomic changes in the society and increased number of working women, and also the conflicting results of studies conducted in relation to the effect of employment on adverse pregnancy out comes, also given the fact that research into occupational hazards has become a priority in determining preventative interventions while identifying mechanisms and causal factors (18), it was decided to conduct a study in this area.

## 2. Objectives

The present study is part of a larger study titled ‘Design of assessment tool and communication model of mother’s lifestyle during pregnancy with low birth weight’, which has been conducted in two stages.

## 3. Patients and Methods

This case-control study was conducted in Tehran in 2012. The data were collected through a researcher-made questionnaire, designed to measure lifestyle with the approach of social determinants of health. Regarding the psychometrics of the questionnaire, face and content validities, criterion validity (19) and construct validity (exploratory factor analysis) were used. The questionnaire contained 132 items in 10 sections: three sections covered general characteristics, pregnancy history and lab test results recorded in the files were also incorporated and seven sections included; physical activity, occupation, nutrition, stress control, self-care, social relationships and inappropriate health behaviors. Cronbach's alpha coefficient confirmed the questionnaire’s high internal consistency (0.76) (20).

In this study, the results related to occupational lifestyle have been presented with 18 items of 5-option Likert style including work environment, duty, working shift, job satisfaction, and employer’s perceived empathy.

In this study, the city of Tehran was first divided into five geographical zones; north, south, east, west and central. Subsequently, from the hospitals in each zone that had a maternity ward, clusters which included one or two government or social security hospitals, were selected according to their delivery rates. Following a review of the literature, the required sample size was determined by considering a 10% prevalence rate for low birth weight, and calculating research variables. The number of items in the measurement tool and key concepts were determined as 3 to 10 samples for each variable (21).

Accordingly, 250 infants with a weight of 2 500 g or less were selected to the case group and 500 infants weighing more than 2 500 g were placed in the control group. The inclusion criteria included:

### 3.1. Mothers

1- Iranian women 15-45 years at a gestational age of 37-42 weeks based on the first day of their last menstruation period (LMP) or sonography, who went to the selected hospitals for their delivery.

2- Lack of problems such as; multiple pregnancy, cardiovascular diseases, diabetes, renal diseases, thyroid disorders, pulmonary diseases, autoimmune disorders, pre-eclampsia, placental abruption, premature rupture of membranes, hepatitis, AIDS and other problems. Mothers who had not used any drugs which could affect the birth weight during pregnancy.

3- Willingness to participate in the research.

### 3.2. Infants

Infants weighing up to 4 500 g, with no known medical problems such as; congenital abnormalities, cardiac or pulmonary diseases, etc.

**Figure 1.**
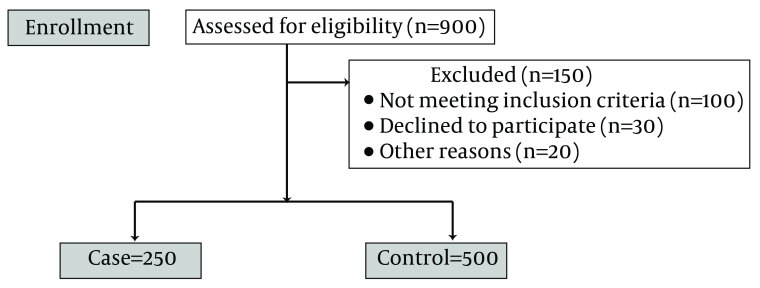
Consort Flow Diagram

After obtaining permission from university and hospital authorities, we presented the required information to the study population and invited them to participate. The questionnaire was then filled out by a team of trained people. First, the researcher selected mothers with inclusion criteria in the delivery room and monitored them until delivery. At the time of delivery, a researcher went to the delivery room, and immediately after delivery, if the infant had no medical problems; congenital disorders, cardiac-pulmonary diseases, etc, and the infant’s weight was 2 500 g or lower using the scale in the delivery room, it was placed in the case group. If it weighed between 2 500 g and 4 500 g, it was placed in the control group (Figure 1). Measurement accuracy of all scales in the delivery rooms was determined by the researcher as follows: a standard weight (control weight of 100 g) was used to calibrate the scale after every 10 samples. Following transference of the mother to the postpartum care unit, women who were in a good condition and willing to participate in the study, were asked to fill out a consent form. Questions related to the patient’s file including; laboratory test results, ultrasound examinations, etc., were completed by the researcher using the mother’s medical file. Another section which included; demographic questions and those related to lifestyle, was filled out by interviewing the mother. 

In this section, the socioeconomic information (family income, education, family size, occupation of spouse and level of spouse’s help with housework) were also collected and included in the study results.

**Figure 2.**
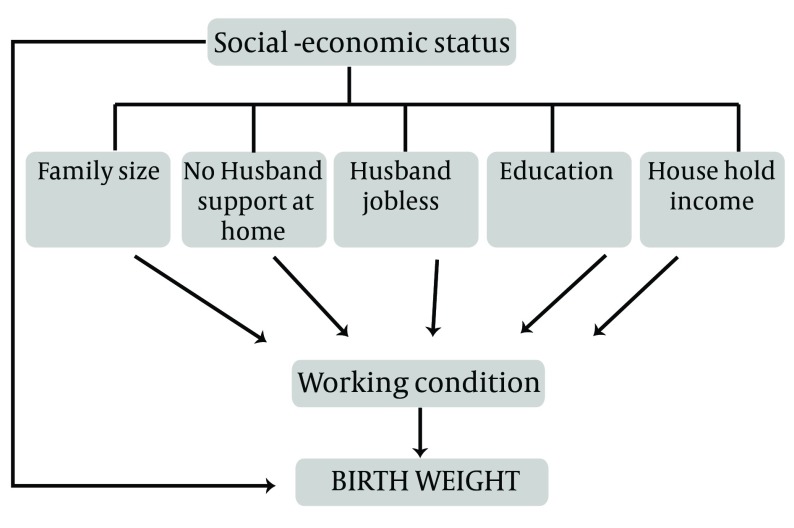
Theoretical Path Model for Effects of Working Condition, Socio-economic Predictors on Birth Weight

In this study, the good fit of a conceptual model of path analysis (Figure 2) was studied to determine the relationship between socioeconomic factors and occupational status of the mother during pregnancy with low body weight. From the results obtained, a logical description of the observed relationships and correlations can be deduced. The SPSS-16 and Lisrel-8.8 software were used for analysis of data with application of path analysis. 

Ethical considerations; the study was conducted following consent given by the chancellors of the University of Tehran, Shahid Beheshti University and the General Department of Social Security for their affiliated hospitals. Moreover, prior to the study, the pregnant women signed an informed consent form after they had been informed of the objectives of the study and were assured that their information would remain confidential and that they could withdraw from the study at any time and also we consider the Patient Privacy.

The study was approved by the Welfare and Rehabilitation Sciences University and the Ethics Committee of the Research Center for the Social Determinants of Health.

## 4. Results

In this study after considering the establishment of the normality assumption in numerical variable by checking with Kolmogorov Smirnov test, it was found that there was no significant difference in terms of; mean age, BMI, pregnancy age and pregnancy intervals found between the case and control groups. However, mothers’ mean weight increase during pregnancy was significantly different in the two groups (P=0.002). In evaluating educational levels, the chance of delivering a low birth weight infant by illiterate mothers was three times higher than in the educated mothers group (P = 0.03, OR = 3.27). Husbands’ occupation and mothers’ employment were among the other factors which were related to low infant birth weight. If the husband was unemployed, the probability of this outcome was 4.5 times higher (P < 0.001, OR = 4.49), in addition, mothers' employment increased the chance of delivering infants with a low birth weight by 5.4 times (P < 0.0001, OR = 5.4). Table 1 shows participants’ individual and social characteristics in both groups. 

**Table 1.** Comparing Some Personal Social Factors of Research Units in the two Groups of Normal Weight and Low Weight Infants 2012

Variables	Normal^a^, Mean ± Sd	LBW, Mean ± Sd	T-test
**Age (years)**	27.34 ± 5.2	27.95 ±5.3	P = 0.13
**Weight before pregnancy(Kg)**	63.07 ± 11.65	63.94 ± 11.47	P = 0.33
**Weight gain (kg) ** ^**b**^	13.92 ± 5.29	12.68 ± 5.06	P = 0.002
**BMI (kg.m^2^)**	24.25 ± 4.14	25.54 ± 4.08	P = 0.35
**HB**	11.98 ± 1.1	11.97 ± 1.9	P = 0.94
**HCT**	36.35 ± 3.6	4.1 ± 36.98	P = 0.22
**Interval of pregnancy(mount)**	5.47 ± 1.17	1.39 ± 5.22	P = 0.06
**Residential density per unit**	26.9 ± 12.65	28.03 ± 12.99	P = 0.25
	**No., (%)**	**No., (%)**	**X^2^**
**Educational ** ^**b**^			P = 0.03,OR=3.273, CI=1.05 - 10.11
illiterate	5 (1)	8 (3.2)	
Literate	495 (99)	242 (5.8)	
**Husbands' job ** ^**b**^			P < 0.0001,OR = 4.49,CI = 2.15 - 9.37
unemployed	12 (2.2)	23 (9.2)	
Employed	488 (97.8)	227 (90.8)	
**Mothers job ** ^**b**^			P < 0.0001, OR = 5.35, CI = 3.34 - 8.58
Employed	29 (5.8)	62 (24.8)	
Housekeeper	471 (94.2)	188 (75.2)	

^a^ Normal weight – infants weighing 2500 g and more ,Low weight- infants weighing less than 2500 g

^b^ significant

To perform path analysis, first, using bivariate analyses, correlations between variables were found. It can be seen that birth weight is directly correlated with mother’s education level, and indirectly (inversely) with working conditions. Also, education and household income are directly and significantly correlated with working conditions. Education is also directly and significantly correlated with income and family size (Table 2). 

**Table 2.** Correlations among Working Condition, Socioeconomic Factors and Birth Weight

	Birth weight	Family size	Household income	education	Working condition
**Birth weight**	1	- 0.15	- 0.009	0.115 ^a^	- 0.336 ^a^
**Family size**		1	0.021	-0.299 ^a^	0.066
**Household income**			1	0.08^a^	0.79 ^a^
**education**				1	0.52 ^a^
**Working condition**					1

^a^ Correlation is significant at the 0.01 level

**Figure 3.**
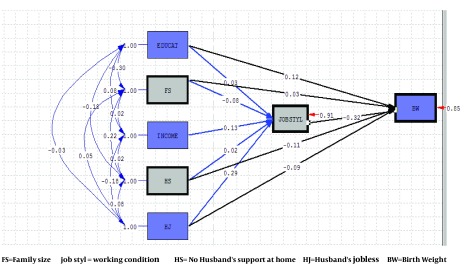
Full Empirical Model (Empirical Path Model for Effects of working condition, socio-economic Predictors on Birth weight)

In path analysis, first the effects of socioeconomic variables (working conditions, income, jobless husband, family size, household income, education, no husband support at home) on birth weight were examined. The results of a preliminary model did not show a good fit (df = 0), and only the parameter of income had a zero path coefficient (β = 0). Thus, this parameter was excluded from the model in direct path on pregnancy outcome, and it was only considered indirectly through its effect on working conditions (Figure 3). After this modification, the model fitted perfectly and its indicators showed high fitness and suitability of the model, and that rational relationships of the variables were based on conceptual model. Accordingly, there was no significant difference between the fitted model and the conceptual model (Table 3). 

**Table 3.** Goodness of Fit Indices for the Model, N=750

Model index	X2	df	p	GFI	CFI	RMSEA
	0.05	1	0.82	1	1	0.000

According to the path diagram, in direct paths, working conditions with β=-0.32 and in indirect paths, income with β = -0.042 had the most effects on birth weight. Unlike other variables, these two variables affected birth weight only through one path (direct or indirect), and the model shows that, due to the negative effects of these variables, mothers whose position is not favorable in terms of these variables, will deliver low birth weight babies.

Unemployment of spouse directly (β = -0.09) and indirectly through affecting working conditions (β = -0.0928) and with overall effect (β = -0.01828) has a negative effect on birth weight. Thus, mothers with unemployed husbands are more likely to have low weight infants. Not having the support and help of the spouse with household chores, also adversely affects birth weight with overall effect (β = -0.1164), and for this reason, these mothers are likely to have low weight babies.

According to the path model, mother’s education level with overall effect (β = 0.1104) positively affects birth weight, and birth weight will be higher with higher level of mother’s education. In this model, 15% of low weight parameter variance is explained by the parameters affecting it. Table 4 presents direct, indirect and the overall effects of the parameters mentioned on birth weight. 

**Table 4.** Path Coefficients for working condition.social-economic-demographic factors on birth weight

Predictor variables	Effects			Model coefficients	t-value	R2	Errorvar
	Direct	Indirect	Total				
**Education**	0.12	-0.0096	0.1104	0.022	3.29		
**Family size ** ^**a**^	0.03	0.0256	0.0556	0.016	0.75		
**Household income**		-0.042	-0.042			0.15	0.34
**No Husband's support at home**	-0.11	-0.0064	-0.1164	- 0.13	3		
**Husband's jobless**	-0.09	-0.0928	-0.1828	-0.27	2.51		
**Working condition**	-0.32	-0.0096	-0.32	-0.012	8.95		

^a^ No significant

## 5. Discussion

In this study, through path analysis, we attempted to find a strong relationship between theoretical and practical issues in research. According to the results, working conditions had the highest direct and negative effect on birth weight, which shows that with more unfavorable working conditions, low birth weight is more likely to occur. Based on the epidemiologic evidence, 5 most common occupational factors including; long working hours, working shifts, lifting loads, standing position, and heavy physical work are involved in the incidence of several pregnancy outcomes such as; preterm birth and LBW, and also as determinants in prenatal and neonatal mortality (10). They also have a role as preventative factors in future adverse effects such as; growth retardation, neurological and congenital defects, hypertension, insulin-dependent diabetes, heart diseases etc (3). In relation to occupation and gender, World Health Organization report (2004) states: despite the lack of information about women’s employment status, especially in low-income countries, hard physical work both at home and in the workplace leads to the incidence of adverse pregnancy outcomes. In a study by Needhammer et al. (2009), it was found that more than 40 hours of work per week and shift work increase risks of low birth weight incidence, small for gestational age (SGA), and preterm labor. They also found that part-time work can be a preventative factor for preterm labor (6). It seems occupational activities that require bending and lifting heavy objects, due to exertion of pressure on the lumbar vertebrae and increased intrauterine pressure, prepare the grounds for incidence of these outcomes (22). In some studies, the reason for increase in these outcomes is considered due to the activity of sympathetic nervous system in the active muscles following occupational activities such as; standing, sitting, and long shifts, resulting in the return of blood from visceral arteries to active muscles, increased sweating and decreased plasma volume and thus, reduced blood perfusion to uterine and placenta arteries (23). Also, hard work, through changes in nutritional status of women plays a role in incidence of these adverse outcomes (22).

Most of the socioeconomic status parameters (household income, mothers’ education, unemployed husband, and no husband support at home) directly or indirectly affected birth weight. But income only indirectly affected birth weight, and through its direct and positive effect on working conditions, followed by negative effect on birth weight, it shows that in poor family income conditions, mother is forced to take jobs with unfavorable conditions, which can negatively affect birth weight. Thus, family income plays an important role in birth weight. This factor is related to mothers’ job and number of prenatal care visits. This finding is in agreement with the results of Zarbakhash Bhari et al. that found low birth weight families were in lower levels of socioeconomic status compared to normal birth weight families (24). Inequality in income can lead to inequality in health, and countries with greater income inequality experience lower life expectancy. Different interpretations have been expressed for inequality of income and health mechanism. For instance, factors like material, structural, behavioral and individual’s lifestyle could be mentioned. Another interpretation is malnutrition and its subsequent infectious diseases, both leading to increased mortality rate in mothers, infants, and children. These deaths are all related to poverty. Hence, improvement in living conditions and increased national income in poor countries leads to improved and increased life expectancy (12).

Another socioeconomic parameter is unemployment of the spouse. This parameter confirms previous findings by its direct and positive effect on mother’s working condition and direct and negative effect on birth weight and the overall negative effect on birth weight. Unemployment of the spouse is accompanied by lower family income, which leads to repeated adverse conditions for the mother and subsequently for her fetus and the newborn. Hawamdeh et al. also believe that material deprivation and economic inequalities caused by poor working conditions such as; poor nutrition, poverty, residential conditions, insufficient income through psycho-social factors of lifestyle and physio-pathological changes have important effects on the incidence of chronic diseases and mental health of people (5). Generally, families in lower socioeconomic level are faced with malnutrition, inadequate care during pregnancy, addiction, smoking and alcohol, successive pregnancies, stress etc., whose consequences may lead to premature labor, intrauterine growth restriction, and low birth weight (24).

Mother’s education, with direct and positive effect on working conditions and birth weight shows that with higher mother’s education level, the birth weight also increases. This finding is in line with the results of many studies. Educated mothers can have better jobs and better conditions and incomes. They also receive prenatal cares and appropriate nutrition, and do not smoke. All these conditions help having a child with normal weight and favorable conditions (25).

Another parameter investigated was lack of spouse’s support and help around home. This parameter, with direct and negative effect on working conditions and birth weight shows that mothers that lack help and support of their spouses at home deliver low birth weight babies. It seems family and social support protects the person against stressful life events through a buffering mechanism, and leads to the well-being of the mother (26). Lack of spouse’s support and help during this period, and continued activities of the mother as before, is accompanied by incidence of unfavorable pregnancy outcome. Several studies have reported the relationship between social and family support and the growth of the infant, and have stated that this factor, through promoting healthy lifestyle, healthy behaviors, and adequate pregnancy cares have a role in improving pregnancy outcome (26, 27).

### 5.1. Conclusion

Since birth weight increases risk of mortality, morbidity, and disability in childhood and in adulthood, it is important to identify its influencing factors. According to the path analysis model, working conditions and socioeconomic status directly and indirectly affect birth weight. Thus, as well as attention to treatment and health care (biological aspect), special attention must also be paid to the mothers’ socioeconomic factors. This is largely possible through attention to socioeconomic problems of mothers and with supportive laws based on their needs.

### 5.2. Strong points

Positive points in this study were equality between study groups in terms of the intervening factors such as gestational age Residual per capita etc.

### 5.3. Limitations

In this study, mothers were interviewed shortly after their delivery; thus, there is the possibility that frustrations resulting from their delivery or pregnancy may have had an influence on their responses. Furthermore, the researchers only studied variables that were amenable to investigation via interviews with the mothers. There may have been other elements as well which affect birth weights that were not taken into consideration in this study. Additional studies should therefore be conducted in this field taking into consideration the previously mentioned factors.
